# Improved Activities of Daily Living With Adjunctive Intravenous Steroids in Bacterial Meningitis: A Nationwide, Population-Based Medical Database Study

**DOI:** 10.7759/cureus.54292

**Published:** 2024-02-16

**Authors:** Tetsuya Akaishi, Kunio Tarasawa, Kiyohide Fushimi, Nobuo Yaegashi, Masashi Aoki, Kenji Fujimori

**Affiliations:** 1 Department of Education and Support for Regional Medicine, Tohoku University Hospital, Sendai, JPN; 2 Department of Health Administration and Policy, Tohoku University Hospital, Sendai, JPN; 3 Department of Health Policy and Informatics, Tokyo Medical and Dental University, Tokyo, JPN; 4 Department of Obstetrics and Gynecology, Tohoku University Hospital, Sendai, JPN; 5 Department of Neurology, Tohoku University Hospital, Sendai, JPN

**Keywords:** intravenous steroids, diagnosis procedure combination (dpc), activities of daily living (adl), barthel index, bacterial meningitis

## Abstract

The benefit of using adjunctive intravenous steroids (IVS) to reduce the neurological sequelae in bacterial meningitis remains inconclusive. This study evaluated the effect of IVS on improving the subsequent Activities of Daily Living (ADL) in bacterial meningitis by analyzing data from a large nationwide administrative medical database in Japan. Data from 1,132 hospitals, covered by the administrative Diagnosis Procedure Combination (DPC) payment system from 2016 to 2022, were evaluated. The ADL levels at admission and discharge were measured using the Barthel Index (BI). Out of the cumulative 47,366,222 patients hospitalized, 8,736 were diagnosed with acute bacterial meningitis and had BI data available. The BI at discharge, adjusted for sex, age, and BI at admission, was significantly better among those treated with IVS (p<0.0001). Exploratory subgroup analyses suggested that this benefit is expected across a broad spectrum of bacterial species. In summary, the use of IVS for improving the subsequent ADL level in bacterial meningitis was suggested.

## Introduction

Acute meningitis is a life-threatening neurological condition seen broadly across countries worldwide. The disease causes more than 200,000 deaths annually worldwide [1-3], and the practice of evidence-based, efficient treatments for each patient with acute meningitis is essential. The benefit of using adjunctive steroids has been actively studied and suggested among patients with bacterial meningitis, but it still remains inconclusive [4,5]. Furthermore, the benefit of using adjunctive steroids for improving subsequent Activities of Daily Living (ADL) levels remains largely unknown. Elucidating the current trends in the use of adjunctive steroids and their impact on subsequent ADL is an important clinical topic. Therefore, the present study investigated a large nationwide administrative medical database to elucidate the effect of using adjunctive steroids on subsequent ADL levels in bacterial meningitis.

## Materials and methods

Study design

**Figure 1:**
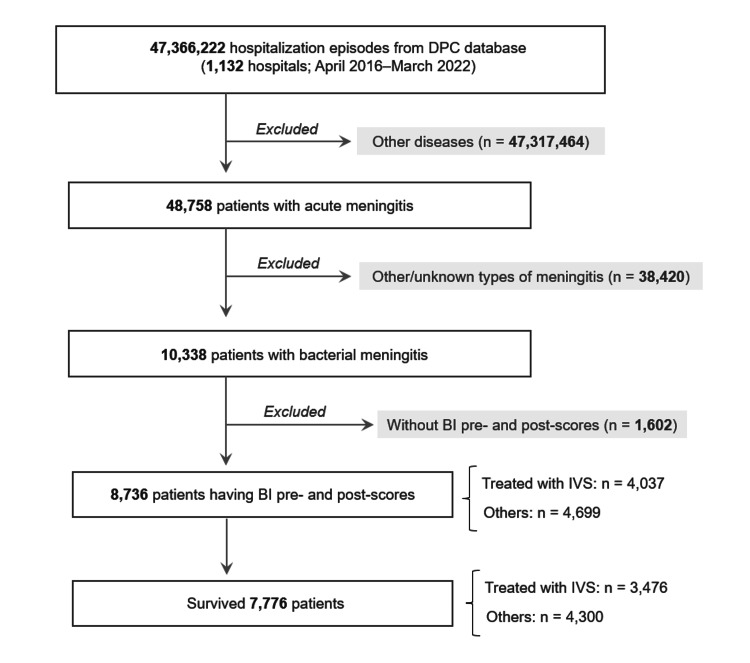
Flow diagram of the study design. From the 47,366,222 hospitalization episodes recorded in the Diagnosis Procedure Combination (DPC) database between April 2016 and May 2022, a cumulative total of 8,736 cases with acute bacterial meningitis and having data on Barthel Index (BI) pre- and post-scores were identified. The effects of adjunctive intravenous steroids on subsequent levels of Activities of Daily Living were evaluated through multivariable analyses among the overall 8,736 patients and among the 7,776 individuals who survived.

This study retrospectively investigated the Japanese nationwide administrative database of the Diagnosis Procedure Combination (DPC) payment system from April 2016 to March 2022. A total of 1,132 hospitals were covered by the system during the study period and agreed to the use of their data for research purposes. Patients with acute bacterial meningitis, who had data on the Barthel Index (BI) scores upon admission (pre-score) and at hospital discharge (post-score), were selected for subsequent statistical analyses [6]. The primary outcome of this study was the eventual ADL level at hospital discharge, measured by the BI score. As another aspect of the primary outcome, the change in BI score during hospitalization (ΔBI = BI post-score - BI pre-score) was also utilized. The study design is illustrated in Figure 1.

Data source

The aforementioned DPC database covers more than 7 million patients treated at DPC-covered hospitals in Japan annually [7,8]. The DPC payment system was initiated by the government in 2003 [9,10]. As of 2023, more than 1,100 hospitals have joined the payment system, including more than 90% of the 81 university hospitals in the country. The database currently encompasses approximately 70% of all annual hospitalization episodes. The diagnosis disease name in each patient's discharge summary is based on the International Statistical Classification of Diseases and Related Health Problems, Tenth Revision (ICD-10) [11]. The investigated disease names were obtained from the registered 'main diseases' in the discharge summary, and not from the provisionally given diagnosis for prescribing medications [12]. Thus, the collected names of diagnoses are reliable and can be considered to reflect the actual disease state in each patient accurately.

Evaluated variables

The diagnostic disease name for each patient was initially searched among the registered 'main diseases', which included the following three entries to capture as many patients with acute bacterial meningitis as possible: (1) principal diagnosis, (2) disease as the primary reason for admission, and (3) disease that required the most medical resources [13]. Two authors (TA and KT) independently reviewed the diagnosis in the discharge summary of each patient for eligibility. Any disagreements regarding eligibility between the two authors were resolved through discussion. In addition to the diagnostic names, age, sex, BI pre-score upon admission, post-score at discharge, and the use of IVS were collected. BI post-scores for those who deceased during the hospital stay were substituted with the worst score of the BI scale (BI score=0).

Statistical analyses

The distributions of demographic and clinical data for continuous variables were described as the median and interquartile range (IQR; 25-75 percentiles). Comparisons of paired continuous variables (BI pre-post data) were performed using the Wilcoxon signed-rank test. Factors possibly influencing the decision to use IVS in bacterial meningitis were investigated using binary logistic regression analysis, adjusting for age, sex, and BI pre-score on admission. The adjusted odds ratio (aOR) and 95% confidence interval (CI) were calculated for each variable. The impact of using IVS on the BI post-score was evaluated using a multiple linear regression model, adjusting for potential covariates. Subgroup analyses, after stratifying the population by identified causative bacterial species and/or mortality, were further performed. A p-value <0.05 was considered statistically significant. The alpha level was not adjusted in the subgroup analyses due to the exploratory nature of the study [14]. Statistical analyses were performed using R Statistical Software version 4.1.3 (R Foundation, Vienna, Austria).

Ethics

This study was approved by the Institutional Review Boards of Tokyo Medical and Dental University (approval number: M2000-788) and Tohoku University Graduate School of Medicine (approval number: 2022-1-441). The review board waived the requirement for written informed consent because patient data were anonymized. All procedures in this study were conducted in accordance with the latest version of the Declaration of Helsinki, as revised in 2013.

## Results

Participants

Of the 47,366,222 hospitalization episodes (from 1,132 hospitals) during the study period, 8,736 cases were identified with acute bacterial meningitis and had both BI pre- and post-scores. Among them, 4,037 (46%) were treated with adjunctive IVS, and 4,699 (54%) were not. Of the 8,736 patients, 960 (11%) died, and 7,776 (89%) survived. The clinical and demographic backgrounds of the overall 8,736 patients were compared by the use of adjunctive IVS (Table 1). The results indicated that adjunctive IVS was more likely to be administered to patients with worse clinical conditions and ADL levels, which could have introduced potential selection bias in IVS use. Therefore, the BI post-score upon hospital admission was decided to be included as an explanatory variable in subsequent multivariable analyses. Sex (p=0.0828) and age (p=0.0189) were also included as explanatory variables.

**Table 1:** Clinical and demographic backgrounds by the use of adjunctive IVS. * Median and interquartile range. P-value was obtained using the Mann-Whitney U test. BI: Barthel Index; IVS: Intravenous steroids.

Characteristics	With IVS (n=4,037)	Without IVS (n=4,699)	P-value
Male, n (%)	2,334 (58%)	2,629 (56%)	0.0828
Age on admission *	65 (46-76) years	65 (38-78) years	0.0189
Mortality, n (%)	561 (14%)	399 (8%)	<0.0001
BI pre-score *	0 (0-50)	10 (0-95)	<0.0001
BI post-score *	75 (0-100)	75 (0-100)	0.8980
ΔBI (= BI post − BI pre) *	0 (0-75)	0 (0-40)	<0.0001

Multivariable analyses among the overall and survived cases

Next, using age, sex, BI pre-score, and the use of IVS as explanatory variables, multiple linear regression analyses for the primary outcome (BI post-score or ΔBI) were performed among the overall 8,736 patients (upper half of Table 2) and among the 7,776 patients who survived (lower half of the table). In both populations, the use of adjunctive IVS was significantly associated with better ADL outcomes (i.e., higher BI post-score and larger ΔBI).

**Table 2:** Multiple linear regression analyses for the subsequent ADL outcomes. ADL: Activities of Daily Living; BI: Barthel Index; IVS: Intravenous steroids; VIF: Variance inflation factor.

Characteristics	Std β	t	P-value	VIF
Outcome: BI at discharge (post-score); population: overall 8,736 patients				
Sex (male)	0.0067	0.73	0.4663	1.002
Age	-0.0870	-9.22	<0.0001	1.065
BI on admission	0.4965	52.30	<0.0001	1.079
Use of IVS	0.0712	7.73	<0.0001	1.017
Outcome: ΔBI (= BI post − BI pre); population: overall 8,736 patients				
Sex (male)	0.0084	0.88	0.3794	1.002
Age	-0.0888	-9.00	<0.0001	1.065
BI on admission	-0.4473	-45.05	<0.0001	1.079
Use of IVS	0.0773	8.02	<0.0001	1.017
Outcome: BI at discharge (post-score); population: survived 7,776 patients				
Sex (male)	0.0254	2.62	0.0089	1.002
Age	-0.0124	-1.25	0.2131	1.055
BI on admission	0.5171	51.58	<0.0001	1.069
Use of IVS	0.1165	11.91	<0.0001	1.018
Outcome: ΔBI (= BI post − BI pre); population: survived 7,776 patients				
Sex (male)	0.0257	2.62	0.0089	1.002
Age	-0.0126	-1.25	0.2131	1.055
BI on admission	-0.4755	-46.86	<0.0001	1.069
Use of IVS	0.1179	11.91	<0.0001	1.018

Subgroup analyses by the identified bacterial species

Finally, subgroup analyses by the identified bacterial species for the effect of adjunctive IVS on the ADL outcome (ΔBI) were performed among the overall patients, regardless of survival (Table 3). In each subgroup of bacterial species, age, sex, BI pre-score, and the use of IVS were used as explanatory variables. A significant effect of using IVS to improve the ADL outcome was observed in bacterial meningitis caused by Streptococcus pneumoniae (n=1,180; p<0.0001), methicillin-resistant Staphylococcus aureus (n=523; p=0.0086), and non-pneumococcal streptococci (n=419; p=0.0265). Meanwhile, a negative impact of using IVS on the subsequent ADL outcome was suggested in Pseudomonas aeruginosa meningitis, although the sample size was too small for statistical significance determination (n=53; p=0.1883).

**Table 3:** Subgroup analyses of IVS for the Barthel Index change (ΔBI) by the bacterial species. The statistics shown are the results of multiple linear regression analyses. The change in BI score during hospitalization (ΔBI) was used as the dependent variable. Age, sex, BI pre-score, and the use of IVS were used as explanatory variables in each subgroup. The analyses were performed among all patients with bacterial meningitis, irrespective of subsequent survival. IVS: Intravenous steroids; MRSA: Methicillin-resistant *Staphylococcus aureus* * p<0.05, ** p<0.01, *** p<0.001

Bacterial species	Statistics for the use of IVS			
	n	Std β	t	P-value
Klebsiella pneumoniae	84	0.0142	0.14	0.8919
Staphylococcus aureus	257	–0.0209	–0.37	0.7118
Listeria monocytogenes	259	0.0258	0.50	0.6179
Escherichia coli	112	0.0563	0.61	0.5399
Streptococcus pneumoniae	1,180	0.1003	3.93	<0.0001 ***
Neisseria meningitidis	76	0.0869	0.84	0.4060
Pseudomonas aeruginosa	53	-0.1774	-1.33	0.1883
Nonpneumococcal Streptococci	419	0.0980	2.23	0.0265 *
Haemophilus influenzae	54	0.1482	1.05	0.2997
MRSA	523	0.1013	2.64	0.0086 **

## Discussion

In the present study, using the nationwide administrative DPC database containing a large number of patients with acute bacterial meningitis, the effect of using adjunctive IVS on subsequent ADL levels at discharge was evaluated among hospitalized patients with the condition. The obtained results indicated that the use of adjunctive IVS was associated with better ADL levels at discharge among the overall cases of bacterial meningitis. Furthermore, the benefit of using adjunctive IVS for facilitating recovery of ADL in bacterial meningitis was suggested to be present across a wide spectrum of bacterial species. Overall, the results indicated that adjunctive IVS may be beneficial for patients with bacterial meningitis in improving subsequent ADL levels. Meanwhile, although the sample size was not large enough to conclusively determine, the use of adjunctive IVS may be better avoided in patients with bacterial meningitis caused by *Pseudomonas aeruginosa*. Further studies with larger numbers of patients are needed to determine the risks of using steroids in *Pseudomonas aeruginosa* meningitis.

When considering the benefit of using IVS in patients with meningitis, it is advisable to separately discuss the following two purposes: (1) reducing mortality, and (2) reducing neurological sequelae and improving subsequent ADL among survivors. A recent meta-analysis suggested the potential benefit of using adjunctive steroids in bacterial meningitis for reducing neurological sequelae, although the mortality rate was not reduced by adjunctive IVS [15]. The finding of reduced neurological sequelae caused by acute bacterial meningitis among those treated with steroids is compatible with the present study. In contrast, previous studies have expressed negative views on the use of adjunctive IVS in other non-bacterial types of meningitis [16-18], especially with the expectation of reducing subsequent neurological sequelae. The administration of adjunctive IVS should be carefully considered based on the expected culprit pathogen and/or condition in each patient with acute meningitis.

This study has several limitations. First, due to the nature of the DPC database, the present study could not utilize laboratory data, cerebrospinal fluid culture yields, or gram staining results of the cerebrospinal fluid for each patient. Therefore, causative pathogens were identified from the registered main disease names. Another limitation of this study is that most of the evaluated subjects were of Asian ancestry, and the generalizability of the obtained findings to other races remains uncertain. In a meta-analysis, the benefit of using adjunctive steroids in bacterial meningitis to reduce neurological sequelae was suggested to differ between high-income and low-income countries [15], indicating that the mechanism of adjunctive IVS in reducing neurological sequelae in bacterial meningitis may depend on environmental factors such as patient background, other medical resources, and co-administered antibiotics. Another limitation is that data regarding the daily dose of IVS and the duration of its use in each patient were not included among the explanatory variables in this study. Finally, due to the retrospective nature of this study, the possibility of selection bias in deciding the use of IVS remains, even though we adjusted for age and baseline ADL level. Additionally, the inclusion of patients with available BI data may introduce selection bias, as patients with more severe illness or poor functional status may be less likely to have complete BI assessments. Future prospective studies with detailed clinical data and standardized protocols are needed to corroborate the obtained findings and further elucidate the role of IVS in the management of bacterial meningitis.

## Conclusions

The present study demonstrated the potential benefit of adjunctive IVS in patients with bacterial meningitis for facilitating recovery in ADL levels. This benefit is expected across a broad spectrum of bacterial species, including *Streptococcus pneumoniae*, non-pneumococcal streptococci, and MRSA. Meanwhile, adjunctive IVS may worsen the subsequent ADL level in cases of *Pseudomonas aeruginosa* meningitis. Further studies are required to conclusively determine this outcome.
